# Influence of the Backward Fall Technique on the Sagittal Linear Acceleration of the Head during a Fall

**DOI:** 10.3390/ijerph19020753

**Published:** 2022-01-10

**Authors:** Andrzej Mroczkowski

**Affiliations:** Department of Sports and Health Promotion, University of Zielona Góra, 65-417 Zielona Góra, Poland; mroczkowskian@gmail.com

**Keywords:** falls, injury prevention, biomechanics of a fall, public health, kinesiology, martial arts, sport, health education, ergonomics

## Abstract

(1) Background: This research aimed to determine the effect of the backward fall technique on the sagittal linear acceleration of the head in students training in different sports. (2) Methods: The study involved 41 students divided into two study groups. Group A included 19 students training in martial arts who practised falls with side aligning of the body. Group B included 22 handball players who practised falls performed in a way similar to a gymnastic backward roll. A rotating training simulator (RTS) was used to force falls, and Wiva ^®^ Science apparatus was used to assess acceleration. (3) Results: Significant changes in head acceleration were only obtained between immediate fall tests (IFTs) and forced fall tests (FFTs) in group B. Significant differences were noted between groups for the IFT and FFT. Greater changes in head acceleration were noted in group B. (4) Conclusions: Smaller changes in head acceleration in group A students indicate a lower susceptibility to head, pelvic and cervical spine injuries in falls performed backward with side aligning of the body. This technique in group A limited the differences in head acceleration between IFTs and FFTs. Negative acceleration values obtained in group B confirmed that the head may suffer a moment of force, tilting it backwards, but then forward when the buttocks hit the ground.

## 1. Introduction

According to reports by the World Health Organization (WHO), falls are the second most common cause of unintentional deaths worldwide, with only road accidents being more frequent. ‘Fall’ is defined by the WHO as an event which results in a person coming to rest inadvertently on the ground or floor, or other lower level [1,2]. A fall can be fatal as well as lead to serious injuries. Falls are especially dangerous for the elderly due to possible problems with bone fusion. Fractures of the limb bones and pelvis are dangerous, but hitting the head against the ground in a fall is particularly dangerous and may result in fatalities [3]. Falls may also lead to dangerous injuries of the cervical spine [4,5]. There are scientific reports stating that school education often does not develop proper motor habits that could reduce the susceptibility to injuries of children and adolescents during a fall [6,7]. Proper development of these motor habits could reduce this susceptibility in children, adolescents and the elderly.

A great many researchers are concerned with the prevention of falls. Falls can be reduced by the elimination of external factors present during a fall or through research on how to improve people’s reactions to disturbances in their balance [8,9]. Accelerating treadmills are usually employed to analyse people’s reactions to fall-generating forces, leading them to losing their balance [10]. It is also possible to utilise platforms [11] and foot-clamps [12,13] to examine backward falls.

It is obvious that a fall at certain values of forces causing it is inevitable. However, if the forces are not too great, a defensive human response is possible, which can reduce injuries during a fall. Therefore, it is sensible to study human defensive reactions during a fall [14,15]. The most commonly used is the division of falls according to their direction into forward and backward falls [16]. Research into the biomechanics of falls is interesting, both for human applications as well as, as scientific reports suggest, for other useful solutions in the design and functionality of humanoid robots. Such research may contribute to reducing the susceptibility to damage of both humans as well as humanoid robots during a fall [17,18,19]. The research included in this article only looked at falling backwards. In the description of this fall, the division was made into the technique of a fall performed in a way similar to a gymnastic backward roll, and a fall performed backward with side aligning of the body [14,15,20]. There are scientific studies justifying the correctness of these techniques in biomechanical aspects [21,22].

Scientists are trying to create the conditions for diagnosing movement habits during a fall. A fall in real conditions may be dangerous to health; the problem is thus to create conditions under which motor habits could be tested without exposing the subjects to injuries. For this purpose, non-apparatus tests were developed to study movement habits when falling backwards. Backward falls are especially dangerous due to their directions [23]. Previously designed tests have been developed for a fall technique similar to a gymnastic backward roll [15,24,25,26]. These tests are easy to perform, but the disadvantage is that the tested falls are not induced by an external force. A better form in this respect is the use of a rotating training simulator (RTS), which forces a fall using inertia. This device can diagnose various types of falls [14,20,27]. The design of RTS is limited to examining adults with high levels of physical fitness. The device is intended mainly for people for whom falls are a frequent event in connection with a sports discipline or work performed, e.g., in uniformed services.

Some research inspiration in the article was the case of a student who complained of the pain in the cervical spine after falling backwards using an RTS. The student did not hit his head against the ground during the fall, but the fall itself was not performed in accordance with the biomechanical recommendations, i.e., an appropriate bending angle in the knee joints and the torso inclination angle relative to the fall ground [21]. In laboratory conditions, mattresses are used, which significantly reduce the acceleration values acting on the head during the entire fall. It would be important to examine the head acceleration figures during falls with the use of an RTS, which would be a certain diagnosis of the accelerations that may occur in real fall conditions [21].

Detailed analysis of the forces causing head acceleration during a fall is difficult because it is a result of the forces generated by the muscles, the forces generated by the contact of body segments with the ground during the fall, and the forces generated by the curvilinear motion within the gravitational field. There are no detailed biomechanical studies on this topic. Biomechanical analysis of the forces acting on the head and resulting from the forces generated by hitting the buttocks against the ground was developed by Mroczkowski [21]. This analysis shows that the effective returns of the resultant force vector inducing acceleration during the fall may be different and cause the head to tilt both back and forth. The information given about the acceleration of the head throughout the fall may give some information as to the susceptibility to damage of other parts of the body linked in the biokinematic chain, in particular, the pelvis and cervical spine.

The main criterion of the research in this article was to determine the value of linear acceleration of the head in the sagittal plane during a fall. These acceleration data were considered to be most important in assessing the risk of hitting the head against the ground during a fall, similarly recognised by other authors [28]. At the same time, based on previous research with the use of RTS and biomechanical studies, it seemed probable that the achieved acceleration values would depend on the fall technique [21,22]. The main aim of the research undertaken in this article was to investigate changes in the linear acceleration of the head in the sagittal plane during a backward fall in the physical education of students playing handball and in students falling backward with side aligning of the body. The other goal was to check whether the obtained acceleration values were consistent with the predictions resulting from biomechanical studies on the backward fall.

## 2. Materials and Methods

### 2.1. Research Material

**Figure 1:**
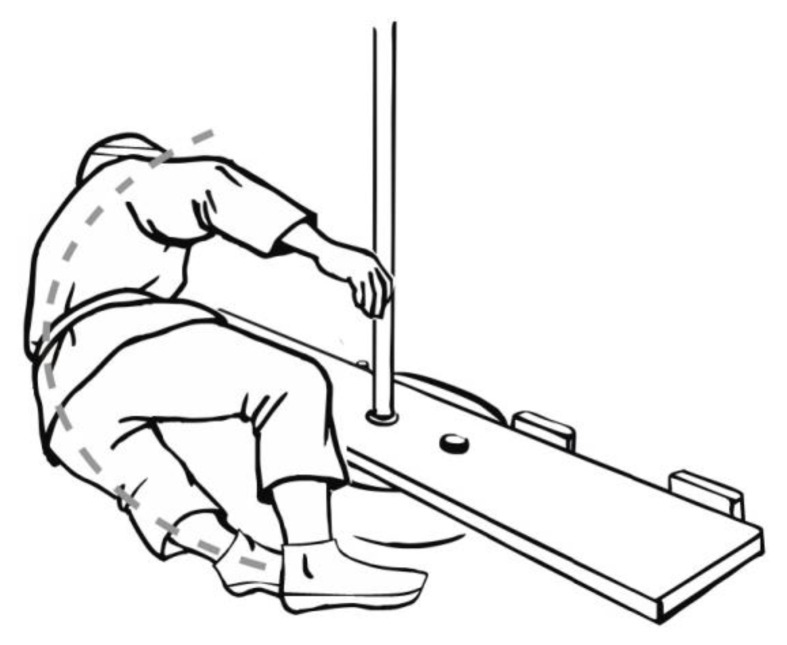
Fall performed backward with side aligning of the body.

**Figure 2:**
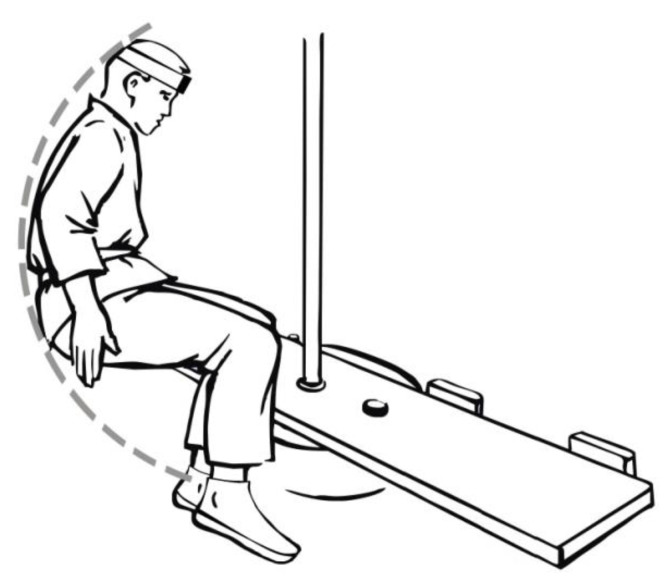
Fall performed in a way similar to a gymnastic backward roll.

A total of 41 physical education students from the university, aged 19–26, qualified for the study. The students were divided into two research groups: A and B. Group A consisted of 19 students who practiced the martial arts of aikido and ju-jitsu. During these classes, they acquired the ability to fall backwards using a technique with side aligning of the body (Figure 1). The students admitted that they were taught the principles of performing this fall technique in their classes. Group B consisted of 22 students who had played handball for at least four years, in sports clubs of the first or second division. During the backwards fall, the students in this group used a technique of the fall performed in a similar way to a gymnastic backward roll (Figure 2). In this fall technique, students typically did not roll over the head in the final stage of the fall, but over the shoulder line [12]. The students in this group stated that in the course of their training, there was no special class explaining the proper execution of this fall technique. In group A, the average height of students was 175 ± 4.5 cm and their weight was 80.9 ± 7.9 kg, whereas in group B, the height was 181 ± 6.2 cm and weight was 82.1 ± 8.4 kg. There were no imposed selection criteria for the study groups in terms of height and weight of students—the selection was random. The research was conducted in the period 2015–2018. All participants gave their informed consent to participate in the research. The study was conducted in accordance with the Declaration of Helsinki, and the protocol was approved by the Commission for Bioethics at the Regional Physicians’ Council in Zielona Góra (4/55/2014).

### 2.2. Research Method

A Wiva^®^ Science motion sensor was used in this study [29], with dimensions of 40 + 45 + 20 mm and a weight of 35 g (Letsense Group, Bologna, Italy). The Wiva^®^ Science sensors consisted of an IMU 9 axis-sensor (accelerometer, 3 axes; gyroscope, 3 axes; and magnetic sensor, 3 axes). The sample rate of the IMU was 100 Hz and the data were transmitted via Bluetooth to a computer, where it was stored using Biomech 2015 software. In the study, the values of linear acceleration in the sagittal plane were analysed. A rotational training simulator (RTS) was used to force a backwards fall. The RTS test method validation procedure for diagnosing a posterior fall was described in [14]. In RTS-induced falls, a person holds on to a pole while standing on a board which is then accelerated to a desired speed. On hearing the sound signal, the person lets go of the pole and the board comes to a halt, which causes inertial forces to induce the person’s fall. Investigators observing falls at lower speeds may exclude students from further participation if the fall could threaten their health. The obtained head acceleration values during the fall were analysed at the speed at which the board was stopped: V3 = 1.5 m/s.

In the experiment, the subjects took part in two tests. In the first test, the students did not try to protect themselves against the fall when the inertial forces that forced them to fall began to act on them. This test was named the “immediate fall test” (IFT). Such a technique of falling is sometimes employed by sports players so that the risk of injury is reduced, or a more favourable decision is obtained from the referee. In the other test, the students only fell when the force causing them to fall was great enough to make them fall. The students tried to keep their balance, thus delaying the fall. This test was named the “forced fall test” (FFT). It can be concluded that for FFTs, the caused fall is consistent with the WHO definition [14,21,22] because students fell inadvertently. At the same time, according to the author of this article, the FFT is more difficult because students delaying the fall have much less time to assume correct body positioning for the fall.

Students who did not make a “hand error” during the fall qualified for the study [15]. “Hand error” reduces the kinetic energy of a fall during the contact of other parts of the torso with the ground [21,30,31]. In the analysis of falls, the “hip error” was not taken into account, because this error only corresponds with the assessment criteria developed a fall using a technique similar to a gymnastic backwards roll, but it does not apply to the technique of falling with a lateral body position [22]. The study participants did not make a “head error” either, which is defined as tilting the head back when changing from vertical to horizontal positions, resulting in the head hitting the ground. In this way, the experiment was limited to examining the acceleration that the head experienced as a result of the impact on it of forces that come from other parts of the body coming into contact with the ground during a fall, e.g., hitting the buttocks.

**Figure 3:**
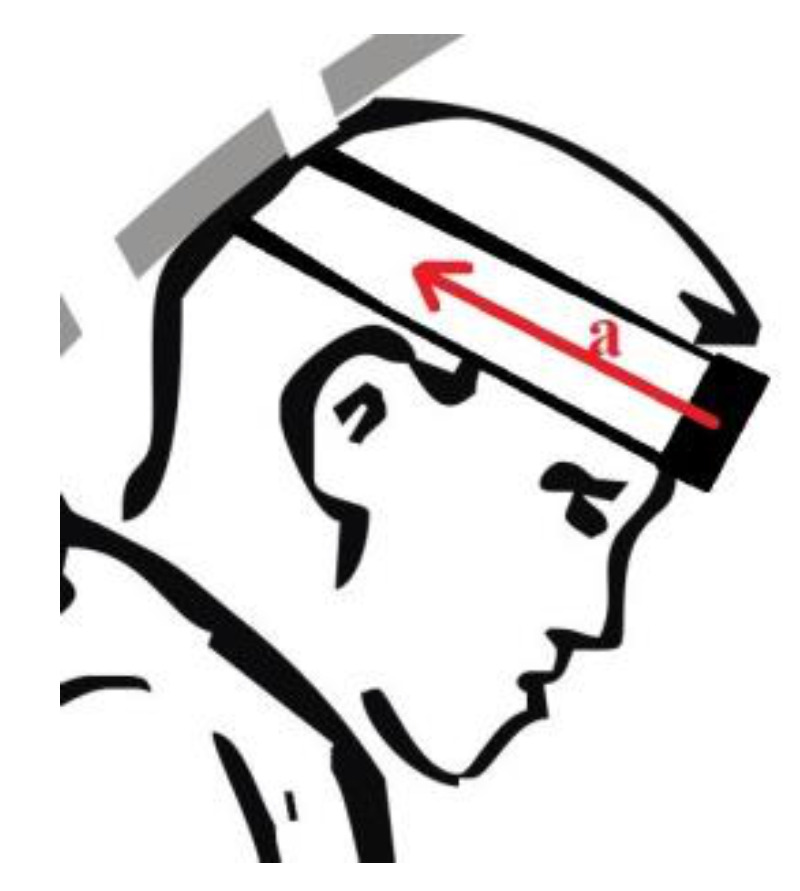
The Wiva ^®^ Science motion sensor mounted to the head to measure acceleration, “a”. The sense of the vector “a” presented in the figures corresponds to its positive values; the opposite direction would correspond to negative values, mainly obtained in group B.

**Figure 4:**
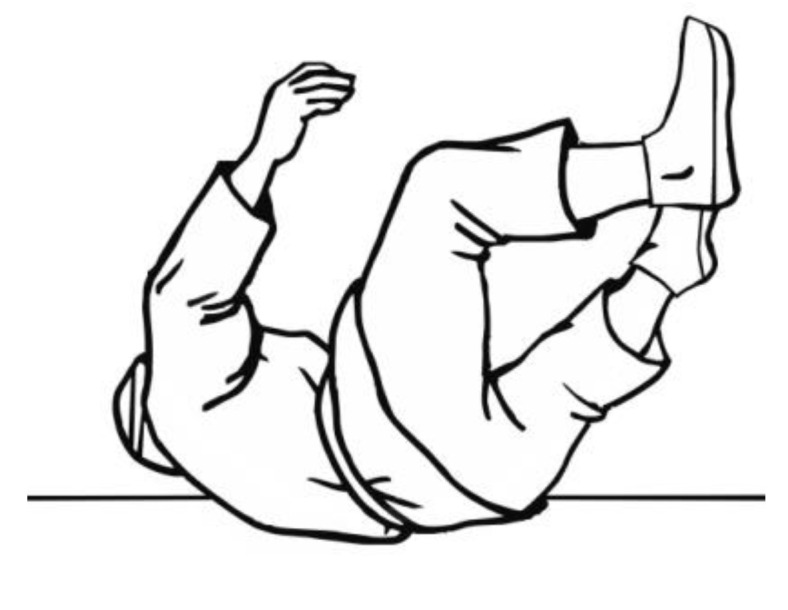
Moment of completion of the head acceleration reading.

Data obtained from the accelerometer of the Wiva ^®^ Science motion sensor were analysed. The sensor was attached to the subjects’ foreheads (Figure 3), and the head acceleration values were analysed from the moment the exerciser lost their balance, leading to a fall (Figure 1 and Figure 2). The sensor was adequately pressed by a band to limit the likelihood of it moving across the forehead during a fall, thus affecting the measurement results. The analysis of the acceleration reading was terminated when the head was not in danger of hitting the ground. Most often, this happened when it was placed parallel to the ground (Figure 4). There were also cases in handball players where the movement of the head was stopped earlier as a result of hitting the ground with the buttocks at a large angle of inclination of the torso relative to the ground of the fall. In such cases, the head was not parallel to the ground due to the transfer of high kinetic energy of the fall through the buttocks. In students from group A, after achieving a parallel position of the head in relation to the ground, the movement resulting from the fall was still performed similarly, as shown in the film [20].

Similar research methods with the use of an RTS with similar research material have already been undertaken [21,22]. They showed that students training in handball or martial arts showed much lower susceptibility to head injuries as a result of hitting the ground compared with students who did not practice any specific sport in sports clubs. The research results in this article were obtained during the tests described above [21,22]. In the course of the research, students worse motion sensors, although not all of them did. Therefore, in the present article, there was a smaller research group of students. This article analyses the values of the linear acceleration of their head in the sagittal plane obtained from the Wiva ^®^ Science motion sensor during a backward fall. This has not been analysed in previous publications.

### 2.3. Statistical Methods

The times of IFTs and FFTs most often differed, both for the same subject and between subjects; therefore, the percentage time to complete the entire exercise was developed from the following formula: kth measurement is k × 100/n% of the time, where n is the number of measurements for a given individual. Other authors [28] carried out a similar analysis for thematically related research. Accelerations were determined for the percentage time points of the exercise execution from 0, 5 and 10, and every 5 to 100, with interpolation. For statistical calculations, only the accelerations assigned to the percentage time points of execution from 0, 5, and 10, and every 5 to 100; common measurements for all subjects were used, and the mean accelerations from all subjects for IFT, FFT and in groups A and B were calculated for them.

In the statistical study, basic characteristics were used, i.e., mean values, n, standard deviations, minimum and maximum values for IFT, FFT, delta IFT, and delta FFT were calculated in each group separately. The statistical methods used were the Student’s *t*-test for dependent variables when comparing IFT with FFT in the same group because the subjects were same. Student’s *t*-tests for independent variables were used for comparisons between groups A and B for IFTs, FFTs, delta IFTs, and delta FFTs. The probability values <0.05 obtained in the tables are in bold. The acceleration values in m/s^2^ considered in the calculations were fed from a sensor that took into account the acceleration due to gravity, g. This means, for example, that a given value of 0.1 sagittal acceleration equalled 0.1 g (0.1 × 9.81 m/s^2^ = 0.981 m/s^2^).

## 3. Results

**Figure 5:**
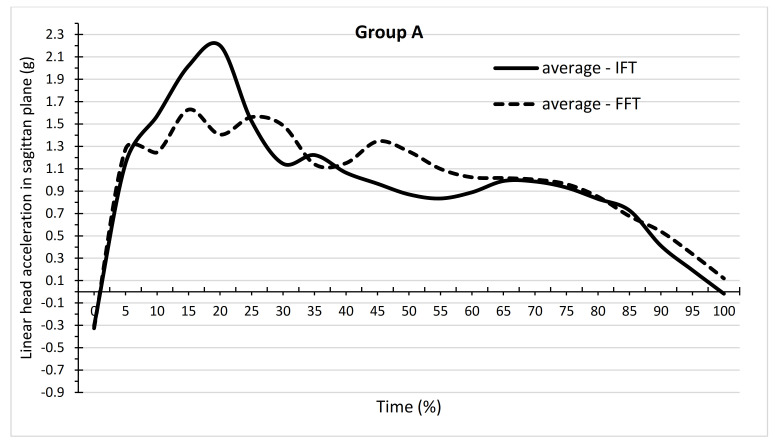
Dependence of mean values of linear head acceleration in the sagittal plane on the time of performing a backward fall in the form of IFT and FFT in group A, which consisted of students training in martial arts.

**Figure 6:**
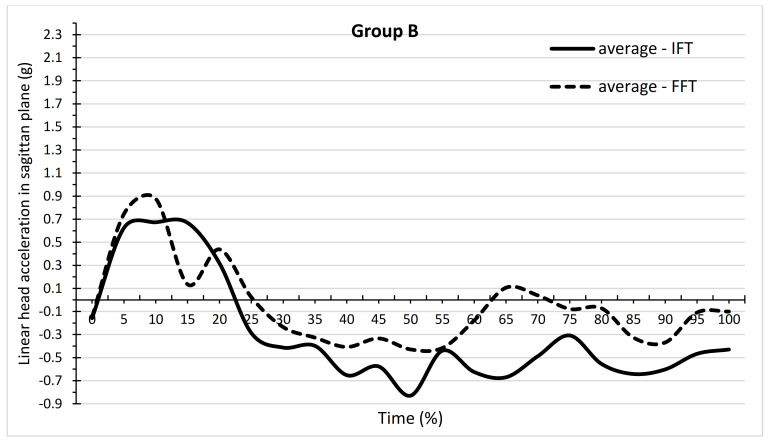
Dependence of mean values of linear head acceleration in the sagittal plane on the time of performing a backward fall in the form of IFT and FFT in group B, which consisted of students who played handball.

Figure 5 and Table 1 show the dependence of mean head acceleration values for IFT and FFT on the time of backward fall in group A. Figure 6 and Table 2 show these relationships for group B. In group A, there are mainly positive values of acceleration, whereas positive and negative values are found in group B. The values of the minimum and maximum accelerations achieved in groups for IFTs and FFTs are shown in Table 1 and Table 2. The values of the maximum accelerations were greater in group A, whereas in group B, the minimum values were greater in absolute terms. Table 1 shows at which time points there were significant differences in the mean values of acceleration between the IFT and FFT in group A. For most time points, the means were not significantly different from each other. IFT differed significantly from FFT at the 20%, 30%, 45%, 50%, and 55% time points, as shown in Table 1 (*p* < 0.05). The means for all the time points, however, did not differ significantly between IFT (0.96) and FFT (0.99). There were more time points in group B, where the mean acceleration values between IFT and FFT differed significantly (Table 2). IFT differed significantly from FFT at the time points 15%, 25%, 50%, 60%, 65%, 70%, 75%, 80%, 85%, 90%, 95%, and 100 (*p* < 0.05). The mean for all time points for IFT (−0.30) differed significantly from the mean for FFT (−0.06).

**Figure 7:**
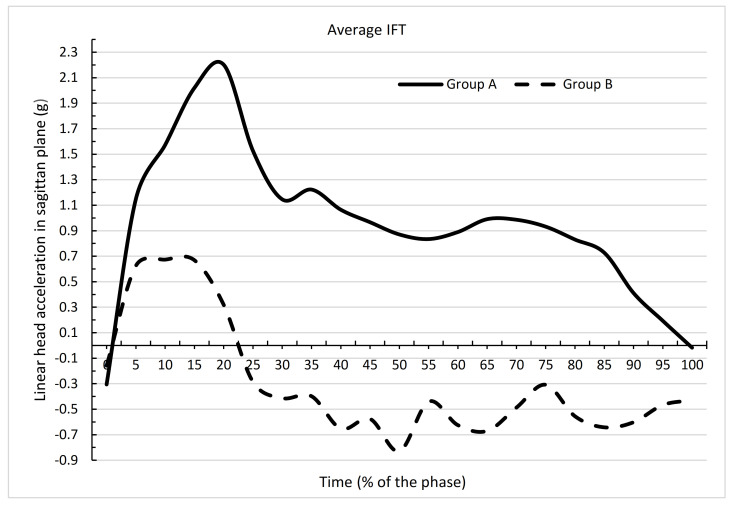
Dependence of mean values of linear head acceleration in the sagittal plane at the time of performing a backward fall in the form of IFT between groups A and B.

**Figure 8:**
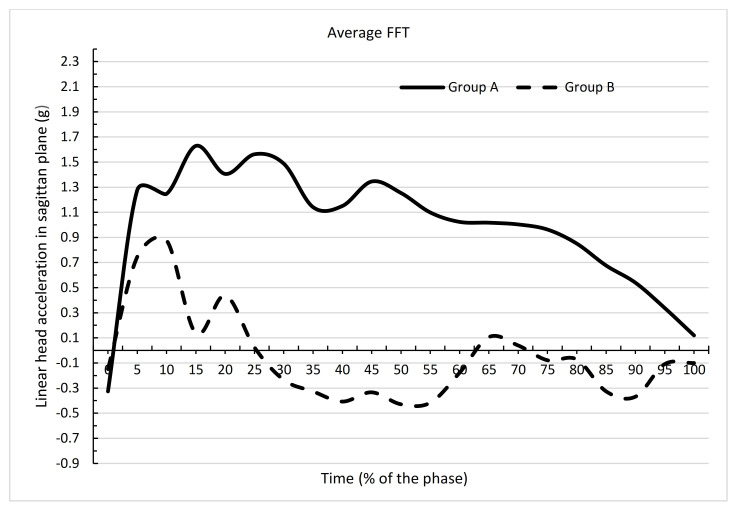
Dependence of mean values of linear head acceleration in the sagittal plane at the time of performing a backward fall in the form of FFT between groups A and B.

Figure 7 and Figure 8 and Table 3 and Table 4 show that the mean acceleration values at nearly all time points exhibited significant differences between the groups for IFT (AIFT and BIFT) and FFT (AFFT and BFFT) performance. IFT differed significantly in groups A and B at all time points except zero. The means in these groups for all time points also differed significantly (*p* = 0.0000). For group A, the mean for IFT was 0.9624, and for group B, the mean IFF was −0.2978. FFT differed significantly between groups A and B at all time points except 0, 10, 95, 100. The means in these groups for all time points also differed significantly. For group A, the mean for FFT was 0.9904, and for group B, the mean for FFT was −0.0554 (*p* = 0.0000). Greater differences were obtained in the acceleration values in group B compared with group A, which was confirmed by greater differences in the adopted minimum and maximum values shown in Table 1 and Table 2. Large differences between the maximum and minimum values will affect the standard deviation. In Table 1, for group A, there was only one standard deviation >1, whereas in Table 2, for group B, there was a standard deviation >1 at many time points.

In Table 5 and Figure 8, values for delta = max (accelerations) − min (accelerations) were calculated for each person separately for IFT and FFT. There were larger delta values for group B than A. The largest delta value was for BIFT, which also has the greatest standard deviation. Table 6 showed significant differences for deltas between the groups for IFT and FFT performance. Between groups A and B, there were greater differences for FFT than IFT.

## 4. Discussion

The results obtained in this article show that in handball players performing a backward fall caused by a horizontal force, significant changes in the linear acceleration of the head in the sagittal plane were obtained between the fall performed in the IFTs and FFTs (Table 2). This was not found in physical education students using the technique of a fall performed backward with side aligning of the body (Table 2). This demonstrates that this habit-acquired fall technique limits the change in head injury susceptibility when falling backwards in more severe FFT fall conditions, a fall as defined by the WHO. At the same time, the lack of change in the susceptibility to head injuries resulted in the lack of changes in susceptibility to damage to other parts of the body connected in the bio-kinematic chain, especially the pelvis and cervical spine.

The author observes that students using the technique of the fall performed backward with side aligning of the body took much less time to adopt the correct body position during the fall than for students using the technique of the fall performed in a way similar to gymnastic backward roll. In the first technique, it is sufficient to twist the torso appropriately because it facilitates the contact with the ground with the lateral position of the lower limb [22]; in the other technique, it is necessary to set the correct bend angle in the knee joints and the torso inclination angle in relation to the fall ground, which guarantees the reduction in the force of hitting the buttocks against the ground [21]. This may explain the significant changes in the acceleration values in group B between IFT and FFT (Table 2). Another fall technique used by students in groups A and B resulted in the fact that the dependencies of head acceleration values on time were different (Table 3 and Table 4) between groups for each type of fall test.

The dependence of the mean values of linear accelerations of the head in the sagittal plane on the time of fall obtained in this article (Figure 5) in group A can be considered as close to the results described by authors who assessed falls performed by judo practitioners [28]. Their fall was not forced by external apparatus, as it was in this study. The difference was also that as the judo practitioners fell during the fall, they hit the floor with their upper limbs, and in this case landed on a mattress. This form of fall is practiced by judo practitioners during fights. It is designed to prevent players from hitting mattresses with a lot of energy with parts of the body more sensitive than the upper limbs. Some of the mechanical energy of the person falling down is transferred to the ground through this impact [31]. The obtained values of acceleration of the heads were slightly lower than those obtained in this article. This can be explained, however, by the fact that during the fall of the judo practitioners, they were not propelled to a certain speed, as was the case with the RTS. The obtained values of acceleration of judo practitioner, similarly to group A in this article, were mostly positive. Comparing the position of individual body segments with the biomechanical assumptions for this form of fall, they can be considered correct [21]. The presented drawings show the appropriate angle of flexion at the knee joint and the angle of the torso in relation to the horizontal to reduce the force of hitting the buttocks against the fall surface.

In the article describing the use of RTS [21], it was found that with the increase in the speed at which the fall is induced, the time needed to properly position the body decreases. As a result, during the fall, the students in group B had straighter legs and a greater angle of inclination of the torso to the ground. Such a position was mainly observed in falls forced on the RTS [21] at V = 1.5 m/s. Biomechanical analysis suggests that with such a setting, it is possible that during a fall, a moment of force tilting the head forward, not just backward, will act on the head. With such an arrangement, a large force is generated acting on the pelvis, which could lead to damage [21,22]. Such positioning of the body segments may not cause the head to hit the ground during a fall, but it will result in a large force transmitted from the buttocks in the bio-kinematic chain through individual parts of the body to the head. Analysis of the film frames obtained during the fall showed that the highest negative head acceleration value was obtained during the tests in group B at the moment of contact of the buttocks with the ground. This ground contact occurred for the exercise completion time percentage points for IFT 47.5 ± 9.7% and for FFT 51.5 ± 10.1%. The negative acceleration values obtained thus confirm the biomechanical assumptions that the head, when hitting the buttocks on the ground, may not only tilt backwards during a fall, but also forwards.

It would be interesting in further studies to determine changes in the value of the linear acceleration of the head in the transverse plane during the impact of the buttocks on the ground. This acceleration could provide more information on the generated force resulting from hitting the buttocks against the ground. At the same time, it should be stated that information on the angle of the torso to the base of the fall in the current research methods concerning the backwards fall is not considered enough, and requires some refinement [21]. In order to more accurately determine the degree of reduction in susceptibility to injury given by a specific fall technique used in the RTS, it would be necessary to measure the acceleration achieved by other parts of the body in addition to the head.

**Figure 9:**
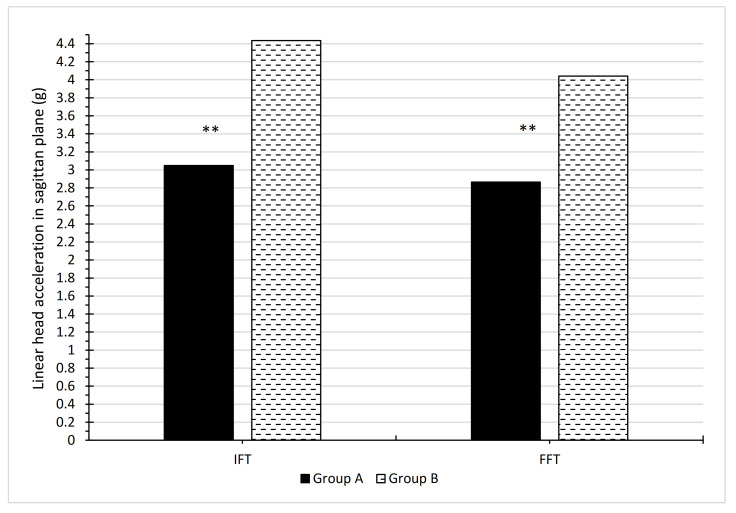
Comparison mean delta values for IFT and FFT between groups A and B (** means a significant difference between the mean values provided at the significance level 0.01).

From the health perspective, it is best for the head not to accelerate rapidly during the fall so as not to generate high inertial forces. When analysing changes in accelerations, the differences were limited to the difference between the maximum and minimum acceleration values (delta). The values of these deltas were definitely higher for subjects in group B as compared with group A (Table 5, Figure 9). At the same time, greater differences were obtained in the adopted values of the minimum and maximum acceleration in group B for both IFT and FFT in Table 1 and Table 2. The smaller changes in head acceleration obtained in physical education students falling with a lateral body position than in handball players also indicate their lower susceptibility to head injuries, and thus to the pelvis and cervical spine as a result of a backward fall caused by a horizontal force.

The results obtained with the use of RTS in these studies are difficult to compare with the results of other researchers, who mainly forced a fall on a standing person. They most often achieved it by applying an external force to a specific segment of the body [16,32], or a sudden tug on the surface on which the examined person was standing [30]. RTS forces a person moving at a certain speed to fall while standing on the board, the sudden stop of which causes an inertia force which induces a fall. The fall is forced here by the exertion of significant forces on the entire human body; therefore, the physical factors causing the fall differed significantly here. The forces with which an RTS can force a fall may be too demanding for people who do not have proper motor habits during a fall, especially the elderly. An RTS is intended mainly for examining adults, in whom a fall is a frequent occurrence in connection with a sports discipline or work performed. This equipment may enable the evaluation of a specific fall technique [31], to diagnose the degree of reduction in susceptibility to bodily injury caused by a given fall technique [14] with a horizontal force causing it. There are scientific reports that the use of a lateral fall technique can prevent hip fractures [33,34,35]. These reports, and the results of research using the RTS, suggest that it would be appropriate to teach this fall technique in school education, because horizontal force is a common cause of fall.

The results obtained in this article do not allow for an unequivocal statement that a fall technique similar to a gymnastic backward roll is incorrect when falling induced by an RTS. It should be assumed that if a group of judo practitioners had been involved in the research, they would have achieved better results compared with the handball players examined in this paper. The worse results of handball players may have been affected by the fact that they had not been subjected to special exercises regarding the principles of safely falling backwards. Descriptions of such principles cannot be found in the literature [36]. The principles of correct falls are, however, described and often constitute a very important part of martial arts classes [37,38].

There are no uniform views on the correct technique of falling. According to Reguli, Senkyr and Vit [39], no ideal falling technique exists. The approach should be adjusted to its prospective use, i.e., a sports discipline to be practised. Footballers, volleyball players, or the general public will not significantly benefit from practicing judo falls to avoid injuries. The biomechanical studies in this area to date suggest that during a fall, the rotational movement of a person on the ground should be similar to the rolling of a car wheel [31]. Parts of the body potentially coming into contact with the ground should be arranged in a circle so as to avoid this. It also seems right that the fall technique should not teach one to roll over their head. As research using an RTS showed, in a group of approximately 800 physical education students, no appropriate movement of the upper limbs was found when falling backwards, which could protect the head in the event of rolling over it [22]. The correct fall technique should depend on the direction of the forces causing it. If the resultant force causing the fall is dominated by the vertical component, the fall is justified with a technique similar to a gymnastic backward roll. An example may be trampoline jumps, where the components of the force causing the fall and velocity are dominant in relation to horizontal forces [40]. In sports where frequent jumps occur, such as volleyball, handball or basketball, this type of fall is justified. However, the performance of vertical jumps during daily physical activity in people who do not practice such sports is rare, especially the elderly. At the same time, a frequent cause of falls is a slip, which is dominated by the horizontal component of the force inducing the fall [14,22,41]. Such a force leads to a fall on the RTS. The results obtained in this paper suggest that the correct technique for such a case is a fall performed backward with side aligning of the body. Biomechanical analysis [22] suggests that a longer total rolling distance in the lateral position of the body during a fall may better distribute stress on individual parts of the body than in a fall similar to that performed similarly to a gymnastic backward roll. Therefore, it seems appropriate to teach the technique of a fall performed backward with side aligning of the body in school education, because horizontal forces are a frequent cause of falls.

## 5. Conclusions

This study compared fall techniques in participants with different sports backgrounds by using an immediate fall test (IFT) and forced fall test (FFT). In students training handball during a posterior fall caused by a horizontal force, significant changes in linear acceleration of the head in the sagittal plane were found between a fall performed when the person does not resist the fall (IFT), and when a person falls inadvertently (FFT). This was not found in physical education students using the technique of a fall performed backward with side aligning of the body. This shows that this fall technique, along with the motor habits acquired, reduces the change in susceptibility to head injuries when falling backwards in more severe conditions (such as FFTs). The obtained lower changes in head acceleration in physical education students falling backward with side aligning of the body position than in students training handball indicate their lower susceptibility to head injuries, and thus to the pelvis and cervical spine, for falling backwards caused by a horizontal force. Thus, it seems appropriate to include the technique of the fall performed backward with side aligning of the body in school education because horizontal force is a frequent cause of falls. The obtained negative acceleration values during the backward fall confirm the biomechanical assumptions that a moment of force may act on the head when the buttocks hit the ground, tilting it not only backward, but also forward. Apparatus for inducing falls, the rotating training simulator (RTS) used in the research, is mainly intended for examining adults, in whom a fall is a frequent occurrence due to the sports discipline or work performed. The forces with which an RTS can force a fall may be too demanding for people who do not have proper motor habits during a fall, especially the elderly. RTS enables researchers to determine the degree of reduction in susceptibility to injury offered by a specific fall technique with the horizontal force of inertia causing it. The results obtained in these studies suggest that it would be appropriate to incorporate the technique of lateral fall in school education because horizontal force is a common cause of falls.

## Figures and Tables

**Table 1:** Basic characteristics (number of observations n, mean, standard deviation SD, max and min) for IFT and FFT for group A. For each time point, the differences between IFT and FFT and Student’s *t*-tests were calculated for dependent variables; it was checked whether this was significantly different from zero.

Time (%)	n(A)	Mean A IFT	SDA IFT	minA IFT	maxA IFT	Mean A FFT	SDA FFT	minA FFT	maxA FFT	Difference (IFT–FFT)	*t*	*p*
0	19	−0.31	0.33	−0.86	0.12	−0.33	0.37	−1.21	0.24	0.02	0.25	0.8023
5	19	1.15	0.43	0.42	1.72	1.28	0.89	−0.02	3.04	−0.13	−0.78	0.4397
10	19	1.57	0.63	0.77	2.90	1.25	0.58	0.33	2.59	0.33	1.94	0.0603
15	19	2.02	0.97	0.69	3.92	1.63	1.04	0.40	3.36	0.39	1.72	0.0938
20	19	2.20	0.88	0.84	3.79	1.41	0.81	0.31	3.39	0.80	4.50	0.0001
25	19	1.53	0.59	0.72	2.66	1.56	0.53	0.61	2.51	−0.04	−0.24	0.8142
30	19	1.15	0.47	0.50	2.20	1.49	0.41	0.93	2.31	−0.34	−2.37	0.0234
35	19	1.22	0.70	0.68	3.23	1.14	0.47	0.49	2.46	0.08	0.40	0.6889
40	19	1.06	0.76	0.19	3.14	1.15	0.54	0.44	2.31	−0.09	−0.41	0.6809
45	19	0.97	0.51	0.17	2.28	1.35	0.58	0.65	2.43	−0.38	−2.66	0.0116
50	19	0.87	0.35	0.15	1.31	1.25	0.50	0.65	2.40	−0.38	−3.54	0.0011
55	19	0.83	0.37	0.03	1.35	1.10	0.38	0.62	2.55	−0.26	−3.24	0.0026
60	19	0.89	0.43	−0.23	1.24	1.02	0.41	0.50	2.48	−0.13	−0.97	0.3376
65	19	0.99	0.54	−0.12	2.12	1.02	0.35	0.61	2.30	−0.03	−0.20	0.8443
70	19	0.99	0.38	0.11	1.58	1.00	0.38	0.33	2.28	−0.02	−0.16	0.8730
75	19	0.93	0.41	0.01	1.39	0.96	0.45	0.19	2.34	−0.03	−0.27	0.7868
80	19	0.83	0.52	−0.13	1.41	0.85	0.44	0.10	2.00	−0.02	−0.19	0.8507
85	19	0.73	0.41	0.09	1.35	0.68	0.43	−0.07	1.39	0.05	0.53	0.6021
90	19	0.41	0.51	−0.51	1.21	0.54	0,57	−0.70	1.42	−0.13	−1.32	0.1942
95	19	0.19	0.42	−0.65	0.69	0.34	0,56	−0.82	0.98	−0.14	−1.95	0.0596
100	19	−0.02	0.31	−0.50	0.39	0.12	0.59	−1.10	1.06	−0.14	−1.56	0.1272
Mean	21	0.96	0.59	0.11	1.91	0.99	0.49	0.15	2.18	−0.03	−0.48	0.6397

**Table 2:** Basic characteristics (number of observations n, mean, standard deviation SD, max and min) for IFT and FFT for group B. For each time point, the differences between IFT and FFT and Student’s *t*-tests were calculated for dependent variables; it was checked whether this was significantly different from zero.

Time (%)	n(B)	Mean B IFT	SDB IFT	minB IFT	maxB IFT	Mean B FFT	SDB FFT	minB FFT	maxB FFT	Difference (IFT-FFT)	*t*	*p*
0	22	−0.16	0.32	−0.80	0.59	−0.15	0.37	−1.46	0.32	−0.01	−0.13	0.9002
5	22	0.62	0.42	−0.12	1.84	0.75	0.52	−0.18	1.74	−0.12	−0.79	0.4319
10	22	0.67	0.46	−0.57	1.41	0.88	0.71	−0.72	2.88	−0.20	−1.31	0.1981
15	22	0.67	0.69	−0.92	1.91	0.14	1.10	−2.87	1.93	0.53	2.52	0.0155
20	22	0.32	0.91	−2.25	1.92	0.44	0.84	−1.16	2.03	−0.12	−0.73	0.4705
25	22	−0.29	1.25	−4.51	1.64	0.02	0.98	−2.03	2.07	−0.31	−2.22	0.0316
30	22	−0.41	1.31	−3.29	2.32	−0. 23	1.00	−2.63	1.39	−0.18	−1.33	0.1911
35	22	−0.40	1.03	−2.42	1.51	−0.33	0.76	−1.49	1.37	−0.07	−0.38	0.7064
40	22	−0.65	1.00	−2.91	1.05	−0.41	0.88	−1.51	2.16	−0.24	−1.28	0.2085
45	22	−0.58	1.10	−3.66	0.68	−0.33	1.12	−2.71	1.87	−0.24	−1.83	0.0740
50	22	−0.83	1.81	−6.85	2.99	−0.43	1.05	−2.05	1.14	−0.40	−3.98	0.0003
55	22	−0.44	1.17	−2.74	2.84	−0.42	0.93	−2.44	0.86	−0.02	−0.31	0.7586
60	22	−0.62	1.25	−4.74	1.49	−0.18	1.18	−2.71	2.08	−0.45	−3.49	0.0011
65	22	−0.67	1.56	−3.77	2.59	0.11	1.52	−2.44	3.12	−0.78	−6.16	0.0000
70	22	−0.49	1.43	−2.32	2.80	0.04	1.29	−2.16	2.47	−0.53	−5.22	0.0000
75	22	−0.31	1.30	−2.13	2.90	−0.08	1.46	−3.19	2.96	−0.23	−2.19	0.0344
80	22	−0.56	0.98	−1.92	1.72	−0.07	1.52	−2.50	3.04	−0.48	−4.93	0.0000
85	22	−0.64	1.09	−3.82	1.27	−0.33	1.25	−2.21	2.28	−0.32	−3.48	0.0012
90	22	−0.60	0.92	−2.55	1.27	−0.37	1.27	−3.12	1.26	−0.23	−2.60	0.0127
95	22	−0.47	0.93	−2.28	1.52	−0.11	0.92	−1.57	1.72	−0.36	−5.18	0.0000
100	22	−0.43	0.71	−1.21	1.14	−0.10	0.64	−1.10	1.02	−0.33	−4.04	0.0002
Mean	21	−0.30	0.46	−2.66	1.78	−0.06	0.36	−2.01	1.89	−0.24	−4.37	0.0003

**Table 3:** Comparison of the sagittal linear acceleration of the head with Student’s *t*-tests for independent variables for IFT between groups A and B.

Time %	n(A)	Mean A IFT	n(B)	Mean B IFT	Difference	*t*	*p*
0	19	−0.3058	22	−0.1582	−0.1476	−1.4671	0.1504
5	19	1.1481	22	0.6240	0.5241	3.9438	0.0003
10	19	1.5723	22	0.6734	0.8989	5.3038	0.0000
15	19	2.0201	22	0.6685	1.3516	5.2061	0.0000
20	19	2.2017	22	0.3189	1.8828	6.6956	0.0000
25	19	1.5264	22	−0.2857	1.8121	5.7865	0.0000
30	19	1.1457	22	−0.4130	1.5587	4.9036	0.0000
35	19	1.2225	22	−0.3981	1.6206	5.8129	0.0000
40	19	1.0647	22	−0.6523	1.170	6.1118	0.0000
45	19	0.9664	22	−0.5764	1.5428	5.6043	0.0000
50	19	0.8705	22	−0.8298	1.7003	4.0229	0.0003
55	19	0.8343	22	−0.4401	1.2743	4.5545	0.0001
60	19	0.8895	22	−0.6248	1.5143	5.0081	0.0000
65	19	0.9904	22	−0.6701	1.6605	4.4106	0.0001
70	19	0.9859	22	−0.4872	1.4732	4.3587	0.0001
75	19	0.9318	22	−0.3080	1.2398	3.9781	0.0003
80	19	0.8305	22	−0.5550	1.3855	5.5299	0.0000
85	19	0.7274	22	−0.6419	1.3693	5.1634	0.0000
90	19	0.4114	22	−0.6020	1.0135	4.2703	0.0001
95	19	0.1933	22	−0.4670	0.6603	2.8623	0.0067
100	19	−0.0174	22	−0.4295	0.4122	2.3446	0.0242
Mean	21	0.9624	21	−0.2978	1.2602	7.6760	0.0000

**Table 4:** Comparison of the sagittal linear acceleration of the head with Student’s *t*-tests for independent variables for FFT between groups A and B.

Time %	n(A)	Mean A FFT	n(B)	Mean B FFT	Difference	*t*	*p*
0	19	−0.3263	22	−0.1486	−0.1777	−1.5300	0.1341
5	19	1.2763	22	0.7450	0.5313	2.3693	0.0229
10	19	1.2469	22	0.8772	0.3698	1.8084	0.0783
15	19	1.6287	22	0.1350	1.4936	4.4549	0.0001
20	19	1.4056	22	0.4385	0.9670	3.7489	0.0006
25	19	1.5620	22	0.0243	1.5377	6.1193	0.0000
30	19	1.4874	22	−0.2348	1.7222	6.9948	0.0000
35	19	1.1408	22	−0.3268	1.4676	7.2863	0.0000
40	19	1.1502	22	−0.4075	1.5578	6.6970	0.0000
45	19	1.3452	22	−0.3338	1.6790	5.8729	0.0000
50	19	1.2539	22	−0.4289	1.6828	6.3553	0.0000
55	19	1.0986	22	−0.4166	1.5152	6.6199	0.0000
60	19	1.0235	22	−0.1775	1.2009	4.2072	0.0001
65	19	1.0172	22	0.1063	0.9110	2.5542	0.0147
70	19	1.0034	22	0.0392	0.9642	3.1325	0.0033
75	19	0.9626	22	−0.0784	1.0410	2.9801	0.0049
80	19	0.8505	22	−0.0720	0.9225	2.5477	0.0149
85	19	0.6761	22	−0.3263	1.0024	3.3145	0.0020
90	19	0.5391	22	−0.3685	0.9077	2.8794	0.0064
95	19	0.3378	22	−0.1091	0.4469	1.8428	0.0730
100	19	0.1195	22	−0.1005	0.2199	1.1301	0.2653
Mean	21	0.9904	21	−0.0554	1.0458	7.8742	0.0000

**Table 5:** Basic characteristics (number of observations N, mean, standard deviation SD, min, max) for the delta variable = max (accelerations) − min (accelerations).

Variable	N	Mean	Minimum	Maximum	Std Deviat.
A IFT	19	3.047947	1.496000	4.570000	0.916093
A FFT	19	2.865105	1.662000	4.600000	0.796852
B IFT	22	4.434295	2.414000	9.694000	1.463410
B FFT	22	4.039909	2.786000	6.164000	0.902734

**Table 6:** Comparison of independent variables, mean delta values for IFT and FFT between groups A and B with Student’s *t*-tests.

	Mean Group A	Mean Group B	*t*	*p*
A IFT vs. B IFT	3.048	4.434	−3.566	0.000976
A FFT vs. B FFT	2.865	4.040	−4.385	0.000085

## Data Availability

The datasets analyzed during the current study are available from the corresponding author on reasonable request.
